# Seroprevalence of Dengue in American Samoa, 2010

**DOI:** 10.3201/eid1902.120464

**Published:** 2013-02

**Authors:** Jennifer Duncombe, Colleen Lau, Philip Weinstein, John Aaskov, Michelle Rourke, Richard Grant, Archie Clements

**Affiliations:** Author affiliations: University of Queensland, Brisbane, Queensland, Australia (J. Duncombe, C. Lau, A. Clements);; University of South Australia, Adelaide, South Australia, Australia (P. Weinstein);; Queensland University of Technology, Brisbane (J. Aaskov);; Australian Army Malaria Institute, Brisbane (M. Rourke, R. Grant)

**Keywords:** dengue, dengue virus, viruses, seroprevalence, IgG, American Samoa

**To the Editor:** Since the 1970s, regular dengue epidemics have caused considerable illness in the Pacific region (*1*). In 2009, an epidemic year, the incidence of reported clinical dengue cases in American Samoa reached 644 cases/100,000 population; in 2010, incidence decreased to 77 cases/100,000 population (*2*). Dengue surveillance in American Samoa is being developed, but the effects of this disease are unknown.

In 2010, blood samples were collected in American Samoa primarily for a leptospirosis seroprevalence study. Samples were also tested for IgG antibodies against dengue virus, and a seroprevalence of 95.6% was observed. We report this finding and advocate improved surveillance and integrated control programs to limit dengue transmission in American Samoa.

A cross-sectional seroprevalence study was conducted during May–July 2010 with the primary aims of identifying risk factors for human leptospirosis and providing an evidence base to direct public health interventions in American Samoa (*3*,*4*). During the study, investigators encountered community concern about dengue and were asked by health authorities to use the remaining collected serum for a dengue seroprevalence study. Amendments to the original human research ethics applications submitted to the American Samoa Institutional Review Board and the University of Queensland Medical Research Ethics Committee (2010000114) were approved.

From the general population of the islands of Tutuila, Aunu’u, and Manu’a, 807 adults were recruited. Households were selected from Tutuila and Aunu’u Islands by using a spatial sampling design to facilitate geospatial analysis (*4*). One adult from each household was asked to volunteer for the study. The small size of villages on the Manu’a Islands meant that spatial sampling was not possible; thus, a convenience sample of volunteers was recruited. A 5-mL blood sample was collected from each participant, information on demographics and risk exposures was obtained by using a standardized questionnaire, and each participant’s primary place of residence was georeferenced.

In October 2011, serum samples from 794 participants 18–87 years of age (median age 39.5 years) were tested at the Australian Army Malaria Institute (Brisbane, Queensland, Australia) for IgG antibodies against dengue virus. Thirteen participants were excluded from the original sample of 807 because of insufficient serum. Samples were screened by using the PanBio Dengue IgG Indirect ELISA Kits (Inverness Medical Innovations, Brisbane, Queensland, Australia) following the manufacturer’s recommendations and protocols. PanBio Dengue IgG Indirect ELISA Kits can detect antibodies to all 4 dengue virus serotypes with a sensitivity of 99.2% and a specificity of 96.2% (*5*). However, these kits cannot identify the specific dengue serotypes responsible for infections. Results were calculated as counts and proportions of PanBio Units (PBU) and allocated a dengue IgG status accordingly: <9.0 PBU was a negative result, 9.0–11.0 PBU was an equivocal result, and >11.0 PBU was a positive result.

Serum samples from 759 (95.6%, 95% CI 93.9%–96.8%) of 794 study participants had IgG antibodies against dengue virus (Table). Seroprevalence for men and women was comparable and did not differ from overall results. As expected, the seropositivity rate was lower among persons 18–25 years of age (89.1%, 95% CI 84.0%–92.6%) because of less time exposed to dengue viruses, and the seropositivity rate was higher among persons 26–40 years of age (99.5%, 95% CI 97.5%–99.9%) than for the overall study population. Despite this study being limited by convenience sampling on the Manu’a Islands, it demonstrates almost universal exposure of sampled adults in American Samoa to dengue viruses.

**Table:** Prevalence of IgG against dengue virus among 794 adults, American Samoa, 2010*

Characteristic	No. positive/no. tested	% Positive (95% CI)
Dengue IgG status		
Negative, PBU <9.0	29/794	3.6 (2.6–5.2)
Equivocal, PBU 9.0–11.0	6/794	0.8 (0.35–1.6)
Positive, PBU >11.0	759/794	95.6 (93.9–96.8)
Sex		
M	402/418	96.2 (93.9–97.6)
F	357/376	94.9 (92.2–96.7)
Age, y		
18–25	179/201	89.1 (84.0–92.6)
26–40	216/217	99.5 (97.5–99.9)
41–53	182/187	97.3 (93.9–98.8)
54–87	182/189	96.3 (92.6–98.2)
Total	NA	NA

*PBU, PanBio units; NA, not applicable.

In the absence of a vaccine, timely and accurate dengue surveillance and consequent public health response is imperative. Current dengue surveillance in American Samoa is passive and relies on clinicians reporting suspected cases to public health authorities. Passive surveillance systems are typically insensitive, and barriers to treatment seeking by residents (distance to health care facility, financial costs, and encouragement from health authorities to stay at home unless symptoms are severe) further reduces their efficiency (*6*). Moreover, passive surveillance systems do not capture asymptomatic infections, which contribute to disease transmission in the community during the viremic stage of illness. Development of an active surveillance system incorporating geographic information systems would enable health authorities to better monitor distribution and intensity of acute infections, identify high-risk areas, and target dengue control activities (*7*).

These preliminary findings should be evaluated by additional study. Further research into dengue seroprevalence in American Samoa should involve identifying dominant and circulating virus serotypes, studying vector population dynamics, investigating dengue exposure among children, exploring environmental risk factors, and integrating these data into active geographically enhanced surveillance systems. In addition, we suggest implementing a sustainable vector control program similar to those undertaken in Vietnam to limit dengue transmission and reduce associated illness in American Samoa (*8*).
